# Implementation of team-based learning (TBL) in a second year medical school course: does prior experience with TBL improve the impact of this pedagogy?

**DOI:** 10.1186/s12909-022-03363-1

**Published:** 2022-04-18

**Authors:** Gonzalo A. Carrasco, Matthew Gentile, Michelle L. Salvatore, Osvaldo J. Lopez, Kathryn C. Behling

**Affiliations:** 1 Department of Biomedical Sciences, Cooper Medical School of Rowan University, 401 S Broadway, Camden, NJ 08103 USA; 2 Office of Medical Education, Cooper Medical School of Rowan University, Camden, NJ 08103 USA; 3 Department of Obstetrics and Gynecology, Cooper University Hospital, Camden, NJ 08103 USA; 4 Department of Medical Sciences, Hackensack Meridian School of Medicine at Seton Hall University, Nutley, NJ 07675 USA; 5 Present address: Independent researcher, Houston, TX 77027 USA; 6 Department of Pathology, Cooper University Hospital, Camden, NJ 08103 USA

**Keywords:** Active learning, Education, Medical, Team-based learning, Undergraduate

## Abstract

**Background:**

We have shown that use of Team-based learning (TBL) in a first-year Infectious Diseases (ID) course improved final examination and course performance. Therefore, we implemented TBL in the second-year Women’s Health (WH) course to improve acquisition of course content. We hypothesized that prior experience with TBL in the first-year of medical school would lead to a strong correlation between TBL performance in the first and second years.

**Methods:**

Our study is a retrospective review of student TBL and final examination performance in the ID and WH courses. The ID course has weekly TBL exercises that cover all course material, while the WH course has one TBL that covers a small portion of the course material. Final examination and TBL individual readiness assurance test (iRAT) scores in the ID and WH courses from three classes (*n* = 226) were obtained with institutional review board approval. Statistical analyses were performed including comparisons of means and correlation studies.

**Results:**

Average WH iRAT scores were significantly higher than ID iRAT scores (9.19 vs. 7.40,*p* < 0.01), and iRAT scores in both courses were highly correlated (*r* = 0.35,*p* < 0.01). When stratifying students based on WH course performance, in struggling students, iRAT but not final examination scores were higher in the WH course than the ID course (8.73 vs. 7.00,*p* < 0.01 and 82.45 vs. 80.51,*p* > 0.05, respectively).

**Conclusions:**

Our results suggest that prior experience with TBL improves TBL iRAT scores, especially in struggling students. Prior TBL experience is also associated with consistent iRAT performance between first- and second-year courses in high performing students.

## Background

Given the rapid rate of change in medical knowledge, pre-clinical medical school curricula are tasked not only with providing medical students with a robust foundation of knowledge, but also the skills necessary to update that knowledge. As such, many schools have turned to active learning strategies which advance student engagement, critical thinking, peer-to-peer teaching, and nurture lifelong learning. One such technique, Team-based learning (TBL™) was developed by Michaelsen in the 1970’s to improve student engagement in a large, university level business class [1]. Since this time, TBL has been used in several educational environments including nursing [2], veterinary medicine [3], dentistry [4], and pharmacy [5], in addition to medical education [6].

In TBL, instructors assign pre-class material to students prior to arriving at the TBL session. Pre-session preparation is assessed at the start of the exercise using an individually completed, closed-book quiz called the individual Readiness Assurance Test (iRAT) [7]. Afterwards, students are assembled into teams of 5–7 students that review the same quiz questions, arriving at consensus answers. This portion of the exercise is called the group or team readiness assurance test (gRAT/tRAT). During this phase, students work together, using peer-to-peer teaching, in order to master the pre-session material. This is followed by a faculty-led, classroom discussion [7, 8].

At our medical school, we first implemented TBL in the Fundamentals course, a first-year, Fall semester course that reviews basic concepts related to biochemistry, genetics, physiology, pharmacology, microbiology and immunology. This was followed by introduction of TBL in the infectious diseases (ID) course, an organ-systems block that takes place during the Spring semester of the first year. From these early experiences, we found that graded TBL exercises encourage students to prepare adequately for the sessions [9], and that weekly TBL exercises in the ID course improve student performance on final examination questions with less course failures [9]. We also found that TBL iRAT scores correlate with final examination performance, and when TBL exercises occur on a weekly basis, such as in our ID course, iRAT scores can be used to identify students at-risk for poor course performance [10].

Given our success with TBL in these first-year courses as well as pre-medical pipeline courses also conducted at our school [11–13], we decided to implement TBL in the second-year Women’s Health (WH) course to improve acquisition of course content related to sexually transmitted infections (STI). We hypothesized that prior student experience with TBL in the first-year of medical school would lead to a strong correlation between TBL performance in the first and second years. To test this hypothesis, we examined student assessment data from the ID and WH courses in three cohorts of students.

## Methods

### Study participants

ID and WH course TBL and final examination data from three cohorts of students (*n* = 226) at Cooper Medical School of Rowan University (Camden, NJ, USA) were obtained and analyzed with Institutional Review Board (IRB) approval from Rowan University (Pro2018000137) for this cohort study. There were 66 students in cohort 1, 78 students in cohort 2, and 88 students in cohort 3. Students included in the study were enrolled in the ID course as first-year medical students, and in the WH course as second-year medical students.

### Study setting

The ID course is a four-week, organ systems-based course that takes place in the second semester of the first year and covers microbiology, virology, and parasitology as they relate to health and human disease. The course includes weekly TBL exercises, as previously described [9], that take place on the first day of each week and review content covered in lectures, case-based learning sessions, and laboratory sessions during the prior week. All course content is covered in the TBL exercises. These exercises contribute 12% to the final course grade (3% for each TBL exercise), with each iRAT worth 1% and each tRAT worth 2%. Application questions are included in these exercises, but they are ungraded. TBL teams are composed of 5–7 students and are designed to maximize diversity in gender, undergraduate institution, and undergraduate grade point average. The iRAT/tRAT is composed of 10 multiple choice questions, and students utilize immediate feedback assessment technique (iF-AT) cards (Einstein Educational Enterprises, Cincinnati, OH) during the tRAT for immediate feedback on whether they have selected the correct answer.

The WH course is a four-week, organ systems-based course that takes place during the second semester of the second year and covers health issues unique to women as well as female-specific reproductive health. In contrast to the ID course, this course has only one TBL that covers a small portion of the course material related to STIs (approximately 8% of the final examination). The assigned prereading material for this TBL is a PowerPoint slide deck that reviews STIs and related antibiotics, and is made available to the students a week in advance. This WH TBL exercise contributes 3% to the final course grade with the iRAT worth 1% and the tRAT worth 2%. Application questions are included in this exercise, but they are ungraded. TBL teams utilized during the first-year ID course are still used in the second-year WH course with few exceptions for students that have either left the class or are returning from a leave of absence. The iRAT/tRAT is composed of 10 multiple choice questions, and students utilize immediate feedback assessment technique (iF-AT) cards during the tRAT for immediate feedback on whether they have selected the correct answer.

At our institution, the office of assessment, in conjunction with course directors, reviews examination questions and related psychometric data to assess validity and reliability. Questions are mapped to individual educational sessions and broad representation of all sessions on the summative course examination is prioritized. Also, goals and objectives of each session are compared to examination questions to verify content validity. A committee comprised of faculty with extensive experience in writing and reviewing medical school examination questions also reviews all examination questions. Psychometric data is used to identify poorly performing questions which may affect examination reliability. Construct validity is also monitored by this committee. The office of assessment compares performance on internal examinations with national level examinations to look for any important associations and also reviews examination psychometric data to ensure that examination questions are meeting internal reliability standards.

### Data analysis

Final examination and TBL iRAT scores in the ID and WH courses from three cohorts of students (*n* = 226) were obtained with Rowan University IRB approval. To study how TBL performance differs in strongly performing and more poorly performing students, we examined TBL iRAT and final examination scores in students performing in the upper and lower 25th percentiles on the WH course final examination. Determination for placement in the upper and lower 25th percentiles was based on all three cohorts, and students failing the course were included in the lower 25th percentile. The purpose of this part of the study was to learn whether iRAT performance can be predictive of students’ overall performance and hence help to identify struggling students for intervention. Mean averages and standard deviations (SD) for iRAT and final examination scores amongst the various study subgroups were calculated, and changes in these scores were evaluated using Kruskal-Wallis One-Way Analysis of Variance (ANOVA) and Newman-Keul’s statistical methods. We also used a Pearson correlation coefficient (r) to assess the strength and direction of the linear association between final examination and iRAT scores. For all analyses, *p* values < 0.05 were considered statistically significant, and p values < 0.01 were considered highly significant. All data were analyzed using IBM SPSS 24 (Armonk, NY).

## Results

Two hundred twenty six students from three consecutive cohorts of medical students in the first-year ID and second-year WH courses were included in this study. Table 1 summarizes the final examination and iRAT scores for these students in the ID and WH courses. Average WH course final examination scores were significantly (*p* < 0.01) higher than those seen in the ID course (88.87 vs. 84.69). WH course iRAT scores were also significantly (*p* < 0.01) higher than the ID course average iRAT scores (9.19 vs. 7.40).

**Table 1:** Average Final Examination and iRAT Scores for Three Consecutive Cohorts of Medical Students in the First Year Infectious Diseases (ID) and Second Year Women’s Health (WH) Courses

	ID Exam Scores	ID iRAT	WH Exam Scores	WH iRAT
**N**	226	226	226	226
**Average**	84.69	7.40	88.87**	9.19^##^
**SD**	6.77	1.04	4.81	1.21

**p < 0.01 compared to ID exam scores; ^##^ *p* < 0.01 compared to ID iRAT scores

Kruskal-Wallis One-Way Analysis of Variance (ANOVA) and Newman-Keul’s statistical methods

Average iRAT scores in the ID course were highly correlated with ID (*r* = 0.45,*p* < 0.01) and WH (*r* = 0.31,*p* < 0.01) final examination scores (Table 2). Importantly, WH course iRAT scores correlated with ID course average iRAT scores (*r* = 0.35,*p* < 0.01) (Table 2). ID final examination scores were also highly correlated with WH course final examination (*r* = 0.47,*p* < 0.01) and iRAT (*r* = 0.37, *p* < 0.01) scores as were WH course iRAT and final examination scores (*r* = 0.33,*p* < 0.01) (Table 2).

**Table 2:** Correlation of Final Examination and iRAT Scores in Three Consecutive Cohorts of Medical Students in the First Year Infectious Diseases (ID) and Second Year Women’s Health (WH) Courses (Pearson correlation coefficient (r))

	ID Exam Scores	ID iRAT	WH Exam Scores	WH iRAT
**ID Exam Scores**	1			
**ID iRAT**	0.45**	1		
**WH Exam Scores**	0.47**	0.31**	1	
**WH iRAT**	0.37**	0.35**	0.33**	1

**p < 0.01

While the ID TBL exercises covered most of the ID course content, only a small portion of WH course content (specifically STIs) is covered in the single WH TBL. Because STIs are first covered in the 2nd week of the ID course, we analyzed student performance on the 3rd TBL of the ID course (iRAT3), which included all the STI-related questions, and studied its possible correlation with TBL iRAT scores in the WH course (Table 3). We found that there was a statistically significant correlation between performance on ID iRAT3 and the ID final examination (*r* = 0.37,*p* < 0.01). We also only found a statistically significant positive correlation between ID iRAT3 and WH iRAT scores (*r* = 0.21,*p* < 0.01, Table 3).

**Table 3:** Correlation of Final Examination and iRAT Scores in Three Consecutive Cohorts of Medical Students in the First Year Infectious Diseases (ID) and Second Year Women’s Health (WH) Courses (Pearson correlation coefficient (r)): Analysis of STIs-related iRAT (iRAT3) in the ID course and the iRAT in the WH course

	ID Exam Scores	ID iRAT3	WH Exam Scores	WH iRAT
**ID Exam Scores**	1			
**ID iRAT3**	0.37**	1		
**WH Exam Scores**	0.47**	0.31**	1	
**WH iRAT**	0.37**	0.21**	0.33**	1

**p < 0.01

To study how TBL performance differs in strongly performing and more poorly performing students, we examined TBL iRAT and final examination scores in students performing in the upper and lower 25th percentiles on the WH course final examination. We found that WH iRAT scores were significantly higher (*p* < 0.01, Table 4) than average ID iRAT scores in students performing in both the upper and lower 25th percentiles. Importantly, the level of improvement in iRAT scores was not significantly (*p* > 0.41) different between these two groups (mean (SD): 1.91(0.79) and 1.73(1.62) for students in the upper and lower 25th percentiles, respectively). Additionally, students performing in the upper 25th percentile in the WH course had significantly (*p* < 0.01) higher final examination scores (*p* < 0.01) as compared to the ID course, while this change was not seen in students performing in the lower 25th percentile (*p* > 0.05, Table 4).

**Table 4:** Final Examination and iRAT Scores for Three Consecutive Cohorts of Medical Students in the First Year Infectious Diseases (ID) and Second Year Women’s Health (WH) Courses: Analysis of Students Performing in the Upper and Lower 25th Percentiles in the WH course

	Upper 25th Percentile Students				Lower 25th Percentile Students			
	ID Exam Scores	iRAT ID	WH Exam Scores	iRAT WH	ID Exam Score	iRAT ID	WH Exam Scores	iRAT WH
N	56	56	56	56	56	56	56	56
Average	88.41	7.77	94.46**	9.68^##^	80.51	7.00	82.45	8.73^##^
SD	5.66	.89	1.53	.51	7.51	1.11	3.42	1.45

**p < 0.01 compared to ID exam scores; ^##^ *p* < 0.01 compared to ID iRAT scores

Kruskal-Wallis One-Way Analysis of Variance (ANOVA) and Newman-Keul’s statistical methods

Average ID iRAT and final examination scores were statistically correlated in students performing in both the upper (*r* = 0.38, *p* < 0.01) and lower 25th (*r* = 0.45, *p* < 0.01) percentiles on the WH course final examination (Table 5). However, we did not detected significant correlations between WH iRAT and final examination scores in these same students (*r* = 0.05, *p* > 0.5 and *r* = 0.19, *p* > 0.5, respectively), likely due to paucity of final examination material covered by the WH STI TBL. Importantly, ID iRAT and WH iRAT scores were highly correlated (*r* = 0.48, *p* < 0.01) only in students that perform in the upper 25th percentile, while we did not find a significant correlation (*r* = 0.22, *p* > 0.5) in students performing in the lower 25th percentile (Table 5). These data seem to indicate that better performing students consistently prepare and therefore perform better in TBL as compared to more poorly performing students.

**Table 5:** Correlation of Final Examination and iRAT Scores in Three Consecutive Cohorts of Medical Students in the First Year Infectious Diseases (ID) and Second Year Women’s Health (WH) Courses: Analysis of Students Performing in the Upper and Lower 25th Percentiles in the WH course (Pearson Correlation Coefficient (r))

	Upper 25th Percentile Students				Lower 25th Percentile Students			
	ID Exam Scores	ID iRAT	WH Exam Scores	WH iRAT	ID Exam Scores	ID iRAT	WH Exam Scores	WH iRAT
**ID Exam Scores**	1				1			
**ID iRAT**	0.38**	1			0.45**	1		
**WH Exam Scores**	0.26	0.019	1		0.34*	0.45**	1	
**WH iRAT**	0.16	0.48**	0.05	1	0.32*	0.22	0.19	1

*p < 0.05; **p < 0.01

## Discussion

In this study, we analyzed the relationship between TBL and final examination performance in a first- (ID) and second- (WH) year course to see how prior experience with TBL influenced later TBL performance. We found that final examination and iRAT scores were significantly higher in the second-year WH course as compared to the first-year ID course (Table 1). Average ID iRAT scores were highly correlated with ID and WH final examination scores, but more importantly, WH iRAT scores correlated with ID average iRAT scores, demonstrating consistent TBL performance between the two courses (Table 2). Because the WH TBL exercises only reviewed STI-related material, which was also covered by the third ID TBL exercise, we examined the relationship between iRAT scores in these two TBL exercises and found a statistically significant positive correlation (Table 3). When examining differences between stronger and more poorly performing students in the WH course, we found that average ID iRAT and final examination scores were statistically correlated in students performing in both the upper and lower 25th percentiles on the WH course final examination. However, no significant correlation between WH iRAT and final examination scores was seen in these same students. Importantly, ID iRAT and WH iRAT scores were highly correlated only in students that performed in the upper 25th percentile in the WH course (Table 5), demonstrating more consistent TBL performance in more strongly performing students.

We found that prior experience with TBL may improve TBL iRAT scores as evidenced by significantly (*p* < 0.01) higher iRAT scores in the second year WH course as compared to the first year ID course, suggesting that these students may have benefitted from prior TBL experience (Table 1). Interestingly, in contrast to another one of our studies where TBL disproportinatley helped students struggling with course content [9], both struggling and highly performing students saw improvement in their iRAT scores in the WH course (Table 4). For students performing in the upper 25th percentile in the WH course, WH final examination scores were also significantly higher than ID final examination scores, suggesting that these students generally perform better in the second year WH course as compared to the ID course, potentially negating the effect of prior TBL experience (Table 4). However, students performing in the lower 25th percentile on the WH final examination did not have significantly higher WH final examination scores than ID final examination scores, and therefore, their higher WH iRAT scores could reflect the effect of prior experience with TBL (Table 4).

Not surprisingly, we found that more strongly performing students have more consistent TBL scores as seen by the high correlation between ID and WH iRAT scores (*r* = 0.48, *p* < 0.01; Table 5). The positive correlation between the iRATs in these two different courses are consistent with recent publications from our group that report a positive correlation between iRAT scores and overall course performance [9, 14]. In this study, it is likely that improved performance in the second year course is due to overall improvement in study habits and better student management of self-directed learning time [15].

We also found that iRAT scores were correlated with, and were therefore predictive of, final examination scores in more poorly performing students in the ID course but not the WH course (Table 5). This was likely due to the paucity of material covered by the WH TBL exercise as compared to the ID course where students participated in weekly TBL exercises each Monday of this four-week course. As we have found in previous studies, consistent weekly TBL exercises have the ability to identify struggling students early in the course to allow for early intervention to ameliorate performance [10]. Although we have reported a highly significant correlation between final examination and iRAT scores for all students in the first year ID course, this correlation was stronger in struggling students as compared to strong performers, highlighting the predictive value of this learning strategy for this population of students [10]. When less TBL exercises are used, such as in the WH course, this benefit is unfortunately lost. On the other hand, some may argue that extensive use of graded TBL exercises, with their associated assessments, may increase student anxiety unnecessarily [15]. Therefore, further investigation is needed to determine the optimal usage of TBL in pre-clinical medical school curricula.

Because this is a single institution study, there are some limitations in generalizability to other institutions. However, because our TBL format is very similar to that described by Michaelsen et al. [16] we feel that for those instiutions that follow this structure, these results could help to guide TBL implementation and use in pre-clinical curricula. Additionally, because of the nature of medical education research and the inability to alter curricula to create informative control groups, we were unable to have the ideal control group which would have consisted of students whose initial participation in TBL exercises occurred in the WH course. The lack of this control group also limits our ability to fully interpret the data generated. However, using other assessment data, including the final examinations in both the ID and WH courses, allowed us to at least partially control for inherent difference between the two courses. Indeed, we found that students performing in the upper 25th percentile in the WH course had significantly higher final examination scores in the WH course, suggesting that these students generally perform better in the second year WH course as compared to the ID course, potentially negating the effect of prior TBL experience (Table 4), while students performing in the lower 25th percentile on the WH final examination did not have significantly higher WH final examination scores, and therefore, their higher WH iRAT scores could reflect the effect of prior experience with TBL (Table 4).

## Conclusion

In summary, our results suggest that prior experience with TBL might improve TBL iRAT scores and is associated with consistent iRAT performance between first and second year courses. Interestingly, in our study, we also found that use of weekly TBL exercises leads to provision of more helpful assessment data to aid in identifying struggling students. The key is to identify the optimal amount of TBL experiences to yield the best educational impact for this active learning activity in pre-medical curricula.

## Data Availability

Raw data for this study is available upon reasonable request to the corresponding author.
